# Reducing Emergency Department Visits for Acute Gastrointestinal Illnesses in North Carolina (USA) by Extending Community Water Service

**DOI:** 10.1289/EHP160

**Published:** 2016-05-20

**Authors:** Nicholas B. DeFelice, Jill E. Johnston, Jacqueline MacDonald Gibson

**Affiliations:** 1 Department of Environmental Health Sciences, Mailman School of Public Health, Columbia University, New York, New York, USA; 2 Division of Environmental Health, Keck School of Medicine, University of Southern California, Los Angeles, California, USA; 3 Department of Environmental Sciences and Engineering, UNC Gillings School of Global Public Health, Chapel Hill, North Carolina, USA

## Abstract

**Background:**

Previous analyses have suggested that unregulated private drinking water wells carry a higher risk of exposure to microbial contamination than regulated community water systems. In North Carolina, ~35% of the state’s population relies on private wells, but the health impact associated with widespread reliance on such unregulated drinking water sources is unknown.

**Objectives:**

We estimated the total number of emergency department visits for acute gastrointestinal illness (AGI) attributable to microbial contamination in private wells in North Carolina per year, the costs of those visits, and the potential health benefits of extending regulated water service to households currently relying on private wells for their drinking water.

**Methods:**

We developed a population intervention model using 2007–2013 data from all 122 North Carolina emergency departments along with microbial contamination data for all 2,120 community water systems and for 16,138 private well water samples collected since 2008.

**Results:**

An estimated 29,400 (95% CI: 26,600, 32,200) emergency department visits per year for acute gastrointestinal illness were attributable to microbial contamination in drinking water, constituting approximately 7.3% (95% CI: 6.6, 7.9%) of all AGI-related visits. Of these attributable cases, 99% (29,200; 95% CI: 26,500, 31,900) were associated with private well contamination. The estimated statewide annual cost of emergency department visits attributable to microbiological contamination of drinking water is 40.2 million USD (95% CI: 2.58 million USD, 193 million USD), of which 39.9 million USD (95% CI: 2.56 million USD, 192 million USD) is estimated to arise from private well contamination. An estimated 2,920 (95% CI: 2,650, 3,190) annual emergency department visits could be prevented by extending community water service to 10% of the population currently relying on private wells.

**Conclusions:**

This research provides new evidence that extending regulated community water service to populations currently relying on private wells may decrease the population burden of acute gastrointestinal illness.

**Citation:**

DeFelice NB, Johnston JE, Gibson JM. 2016. Reducing emergency department visits for acute gastrointestinal illnesses in North Carolina (USA) by extending community water service. Environ Health Perspect 124:1583–1591; http://dx.doi.org/10.1289/EHP160

## Introduction

The introduction of the community water system (CWS) was one of the twentieth century’s most significant public health advances (Cutler and Miller 2005). In the United States, this intervention is credited with decreasing infant, child, and total mortality by 75%, 67%, and 50%, respectively, between 1900 and 1936 (Cutler and Miller 2005). However, despite the potential health benefits provided by CWSs and by decades of investment in expanding drinking water infrastructure, 44.5 million U.S. residents (14% of the population) lack access to a regulated community water supply and instead obtain drinking water from an unregulated source, typically a groundwater well but sometimes a spring or surface water source (Maupin et al. 2014). For regulatory purposes, a domestic water system (DWS) is defined as an individual household well or other residential water system with fewer than 15 connections or serving fewer than 25 people year-round [U.S. Environmental Protection Agency (EPA) 2015]. Among U.S. states, North Carolina has the second-largest population—3.3 million residents (35% of state residents)—relying on DWSs for their drinking water (see Figure S1) (Maupin et al. 2014).

Private wells and other DWSs are not regulated by the U.S. Safe Drinking Water Act (1974) and therefore are not subject to the same level of monitoring as CWSs. To improve the safety and quality of drinking water from DWSs, the North Carolina General Assembly passed a law requiring all counties to institute a private drinking water well permit program by 1 July 2008 (General Assembly of North Carolina 2006). Under this program, all new private wells must be permitted and must undergo water quality testing at the time of construction. However, this program may not be as effective as desired because routine monitoring is not required after the permit is granted, and wells constructed before 2008 are exempted. Furthermore, there are no requirements to treat private well water if contamination is detected.

The magnitude of waterborne disease attributable to contaminants in U.S. private wells is thought to be substantial but is not well quantified. Previous U.S. studies have sought to quantify microbial pathogen concentrations in private wells and in CWSs that use undisinfected groundwater (Allevi et al. 2013; Borchardt et al. 2003; DeSimone and Hamilton 2009; Sandhu et al. 1979; Sworobuk et al. 1987), and a few studies have sought to establish relationships between self-reported health outcomes and microbial contaminant concentrations in drinking water (Borchardt et al. 2012; Heaney et al. 2013; Macler and Merkle 2000; Raina et al. 1999; Uhlmann et al. 2009; Wedgworth and Brown 2013). However, to our knowledge, no U.S. study has provided county-level estimates of the burden of acute gastrointestinal illness (AGI) attributable to microbial contaminants in private wells for an entire state. The limited knowledge of the magnitude of health risks associated with private well contamination suggests that a comprehensive burden of disease assessment could inform future decisions about whether to extend community water service to unserved/underserved areas or to establish other policies to protect the health of those relying on private wells.

To help fill the information gap on waterborne disease risks associated with U.S. private wells and on the potential health benefits of interventions to reduce risks, in this study, we developed a population intervention model (PIM) to quantify AGI risks attributable to microbial contaminants in North Carolina private wells. We focused on AGI because analyses of U.S. waterborne disease outbreak data over the past four decades indicate that AGI was the health outcome of concern in 87.8% of outbreaks (Craun et al. 2010). The PIM method enables not only estimation of current risks but also of potential risk reductions that could be achieved if CWSs were extended to those relying on domestic wells. As described by Hubbard and van der Laan (2008), PIMs are intended to estimate “the difference between a treatment-specific counterfactual population distribution and the actual population distribution of an outcome in the target population of interest.” The PIM approach has been used to estimate the health effects of a range of interventions from reductions in perceived stress to smoking cessation (Ahern et al. 2009; Fleischer et al. 2010). A recent review recommended its use for quantifying the global disease burden associated with poor drinking water quality and lack of sanitation facilities (Clasen et al. 2014). However, this approach has not been used previously to estimate public health risks from contaminated private wells in the United States.

The majority of previous studies of microbial hazards of U.S. private wells quantified microbial contaminant concentrations but did not extend their analyses to estimate the associated health risks. A recent U.S. Geological Survey (USGS) study of ~400 private wells throughout the United States found that 34% were contaminated with total coliform bacteria and 8% were contaminated with *Escherichia coli*. (DeSimone and Hamilton 2009). A prior study in a central North Carolina neighborhood found that 5 of 12 wells tested positive for fecal indicator bacteria, but none of the eight houses connected to a CWS tested positive (Heaney et al. 2013). A study of microbial contaminants in Virginia domestic wells found that 41% of 538 samples tested positive for total coliforms and 10% tested positive for *E. coli* (Allevi et al. 2013). A Wisconsin study found that 28% of 50 private wells tested positive for total coliforms and 8% tested positive for enteric viruses (Borchardt et al. 2003). In Preston County, West Virginia, a study of 155 private wells found that 68% tested positive for total coliform bacteria (Sworobuk et al. 1987). Finally, a study of three rural South Carolina counties randomly sampled 460 private wells (representing ~10% of well users) and found that 85% of samples were positive for total coliforms (Sandhu et al. 1979). These studies suggest that the detection frequency of microbial contaminants is substantially higher in private wells than is currently permitted in CWSs under Safe Drinking Water Act (1974) regulations, which require that no more than 5% and 0% of samples test positive for total coliform and *E. coli* bacteria, respectively, each month.

Very few recent North American studies have sought to link AGI risks to microbial contamination of private drinking water wells (Raina et al. 1999; Uhlmann et al. 2009). A recent cross-sectional case study in Alabama found that drinking water that tested positive for fecal coliforms increased the odds of contracting AGI by a factor of 4.0 [95% confidence interval (CI): 1.3, 14], regardless of whether the water was from a domestic well or a CWS (Wedgworth and Brown 2013). This study also found that 20% of samples from DWSs tested positive for fecal coliforms, a proportion that was 2.5 times higher than samples from households connected to a CWS. In addition, a recent study in British Columbia, Canada, estimated that individuals drinking water from private wells had a 520% increase in AGI risk compared with those supplied with water from CWSs (Uhlmann et al. 2009). These findings suggest that households relying on private wells are exposed to more waterborne pathogens than those served by CWSs and thus may suffer more negative health outcomes than municipally supplied households.

The study reported here applies a PIM approach to estimate the risk and cost of AGI associated with exposure to microbial contaminants in North Carolina private wells. This is the first study to provide such a quantitative, comparative analysis for an entire state at the county level using local health outcome and water quality data to produce population-specific estimates. Application of the PIM method to assess North Carolina waterborne disease risks was enabled by the establishment of two North Carolina databases: one that tracks illnesses reported in every North Carolina emergency department and another that houses all private well water quality sampling data collected through North Carolina’s DWS permitting program. Both databases are the result of laws enacted by the North Carolina General Assembly: a 2004 statute requiring the North Carolina State Health Director to establish the North Carolina Hospital Emergency Surveillance System and obligating all emergency departments to submit electronic records of all visits to the system on a daily basis (General Assembly of North Carolina 2004), and a 2006 law requiring a water quality test at the time of installation for all wells constructed on or after 1 July 2008 (General Assembly of North Carolina 2006). Our results not only identify North Carolina counties that may benefit the most from expanding CWSs but also provide insights into the potential magnitude of the disease burden attributable to microbially contaminated, unregulated private wells in the United States. The method we demonstrate could encourage other states to develop databases similar to those in North Carolina to assess the burden of disease associated with a lack of access to regulated drinking water systems.

## Methods

The PIM approach for estimating the burden of disease attributable to a particular risk factor relies on a causal inference framework that describes the relationship between the current population distribution of exposure to the risk factor and the incidence rate of the health outcome of interest for population groups exposed at different levels (Hubbard and van der Laan 2008). For this analysis, the exposure of interest was microbial contamination of drinking water from CWSs and DWSs, and the health outcome of interest was AGI. The following section describes the data sources used to characterize exposure to microbial contaminants in drinking water and the incidence rate of AGI in North Carolina counties. Next, we describe the mathematics of the PIM approach, followed by the sources of data used to translate the PIM results into estimates of the health costs of AGI attributable to drinking water contamination.

### Data

***Private well water quality data.*** We received monitoring data for all newly constructed private wells for the 60-month period 1 January 2009–31 December 2013 from the North Carolina State Laboratory of Public Health (N. Barros, NC Department of Health and Human Services Environmental Epidemiology Team Leader, e-mail communication, 1 April 2014). The data set included results from tests of 16,138 private wells for total coliforms and *E. coli* (reported as presence/absence) and the county in which the well was located. According to the 2006 new well construction law, “water samples shall be collected from the sample tap at the well or the closest accessible collection point to the water source with a tap capable of being disinfected, provided the sampling point shall precede any water treatment devices” (General Assembly of North Carolina 2006). Therefore, the well sample data did not account for in-home water treatment.

Data were received for 91 of the 100 North Carolina counties. Among these 91 counties, observations were available for each of the 60 months in 70 counties. In the remaining 21 counties, the number of months for which observations were available ranged from 10 to 58. Because of the incomplete temporal coverage of these data, private well water quality in each county was represented as the proportion of all samples collected in the county during the 60-month time period that tested positive for total coliform bacteria. The statistical PIM described below was fitted to data from the 91 counties for which well water quality data were available, but estimates of health impacts of private well contamination were made for all 100 counties on the basis of this statistical model. When estimating health impacts for the nine counties that did not report, we assumed that the prevalence of microbial contaminants equaled the mean prevalence among bordering North Carolina counties. We also performed a sensitivity analysis in which exposure in these counties was assumed to equal the 15th and 85th percentiles of contamination prevalence in the state as a whole (25.6% and 51.0%, respectively) rather than the mean exposure in surrounding counties.

***Community system water quality data.*** The North Carolina Department of Environment and Natural Resources (NCDENR) provided microbial water quality violation data for all 2,120 active North Carolina CWSs from 1 January 2007 to 31 December 2013 (J. Cavalier, NC Department of Environmental Quality, Public Water Supply Section, Environmental Engineer, e-mail communication, 21 March 2014). The data set contained information on monthly violations, which were defined as events wherein > 5% of samples over a 30-day period tested positive for total coliform bacteria, and as acute violations, defined as the presence of *E. coli* in one or more follow-up analyses of samples testing positive for total coliform bacteria (U.S. EPA 1989).

***Population served by water system type.*** The populations served by CWSs and by private wells were determined using annual county population estimates from the U.S. Census (Minnesota Population Center 2011) together with CWS data reported by NCDENR (J. Cavalier, NC Department of Environmental Quality, Public Water Supply Section, Environmental Engineer, e-mail communication, 21 March 2014). We calculated the county-specific population served by CWSs by summing all individual CWS populations within a given county. We assumed those not served by a CWS relied on private wells.

***Emergency department visits for AGI.*** Because most AGI cases are unreported (Roy et al. 2006; Scallan et al. 2011a,b), we used data on emergency department (ED) visits for AGI as a proxy for total AGI incidence. Data on the total number of reported ED visits for AGI between 1 January 2007 and 31 October 2013 were extracted from the North Carolina Disease Event Tracking and Epidemiologic Collection Tool (NC DETECT), which includes records from all 122 EDs in North Carolina (A. Fleischauer, Captain, US Public Health Service, and Career Epidemiology Field Officer, U.S. Centers for Disease Control and Prevention, written communication, 3 March 2014). Owing to potential privacy concerns, all patient identification data other than county of residence were removed, and data were aggregated by month. In keeping with prior research on AGI, records from NC DETECT containing the following World Health Organization’s *International Classification of Diseases, Ninth Revision* (ICD-9), diagnostic codes were retrieved: infectious GI illness (001–009); noninfectious GI illness (558.9); and diarrhea, nausea, and vomiting (787.01–787.03, 787.91) (Colford et al. 2006; Messner et al. 2006; Roy et al. 2006; Tinker et al. 2009). In total, the database contained 2,769,620 ED visits that matched these criteria.

### Population Intervention Model (PIM)

The PIM approach, which is based on modern causal inference theory, was used to estimate monthly AGI ED visits per county attributable to microbially contaminated CWSs and private wells under different exposure scenarios (Hubbard and van der Laan 2008). To implement the PIM, a panel structure natural log–Poisson regression model with a log-person-month offset and temporally autocorrelated errors was fitted to monthly county-level health outcome and water quality data. The model form is as follows:

ln(Y*_i,j_*_/_ *N_i,j_*) = α + β_1_ *C*_CWS_ *__i,j__* + β_2_ *E*_CWS_ *__i,j__* + β_3_ *C*_DWS_ *__i__* + β_4_ *Pov_i_* + β_5_ *ED_i_* + β_6_ *I_i_* + (Σ^^9^^ *__l__* __= 7__ β*_i_* R*_i_*) + (Σ^^20^^ *_m_*_= 10_ β*_m_* t*_m_*) + μ*_j_*, [1]

where *Y_i,j_* is the number of observed AGI ED visits by residents of county *i* during month *j*; *C_CWS,i,j_* is the proportion of the county population in county *i* exposed to a monthly Safe Drinking Water Act (1974) maximum contaminant level (MCL) violation as defined under the Total Coliform Rule (U.S. EPA 1989) during month *j* (determined by assuming that all customers of a CWS with a monthly MCL violation were exposed); *E_CWSi,j_* is the proportion of the county population exposed to an acute MCL violation; *C_DWSi_* is the proportion of the population in county *i* potentially exposed to total coliform bacteria via a private well (determined by multiplying the fraction of wells testing positive by the proportion of the county population relying on private wells); *R_i_* indicates the region of the state in which the county is located (Coastal Plain, Piedmont, or Mountain); *t_m_* is an indicator variable for month of the year; *N_i,j_* is the county population; *Pov_i_* is the proportion of the county population living in poverty; *ED_i_* is an indicator variable representing whether county *i* contains an ED; and *I_i_* is a binary variable representing whether the proportion of the county that is uninsured exceeds the statewide mean uninsured rate of 16% (= 1 for counties exceeding the statewide mean). The first-order autoregressive error term is represented as *μ_j_*, where

μ*_j_* = ϕ μ*_j_*_– 1_ + ε*_j_,* [2]

and the ε*_j_* are assumed to be independent with a mean of zero. Annual county population estimates were obtained from the NC Office of State Budget and Management (2015). Poverty and health insurance coverage data were obtained from the 2010 U.S. Census (http://www.nhgis.org). Region was used as an indicator variable to reflect distinct differences in landform and geology that may affect water quality, as indicated in previous studies (Markewich et al. 1990). The model was fitted to data for the time period 1 January 2007—31 October 2013 to maximize use of the ED visit data. Regression models were fitted using STATA IC 12 (StataCorp LP).

The fully parameterized, fitted regression model (Equation 1) was used to estimate the observed AGI cases in each county attributable to microbial contamination of CWSs and private wells. The expected number of AGI cases for each county was estimated under both current exposure conditions and multiple counterfactual scenarios in which different proportions of the population relying on private wells were provided with a connection to the nearest CWS. Risks under actual conditions were computed by using all parameters in the regression model to estimate *Y_i,j_* (the mean estimated number of AGI ED visits in county *i* during month *j*) under the current exposure scenario. Risks under counterfactual scenarios were computed in the same manner under multiple different scenarios: *a*) zero exposure to contaminants in drinking water (in either CWSs or private wells); *b*) zero exposure to contaminants in CWSs; *c*) zero exposure to contaminants in private wells; *d*) connection of 10% of the population currently relying on private wells to the nearest CWS. *Y_i,j–counterfactual_* for each county and month was estimated under each counterfactual exposure scenario by changing the relevant independent variables in Equation 1 (e.g., for scenario *b*, *C_CWSi,j_* = 0) to predict the number of AGI cases under that scenario for each county and each month. The natural log change in AGI ED visits given the changes in exposure under each counterfactual scenario was then computed by subtracting the estimated natural log of the counterfactual case rate from the mean regression model estimate of current log of the case rate:


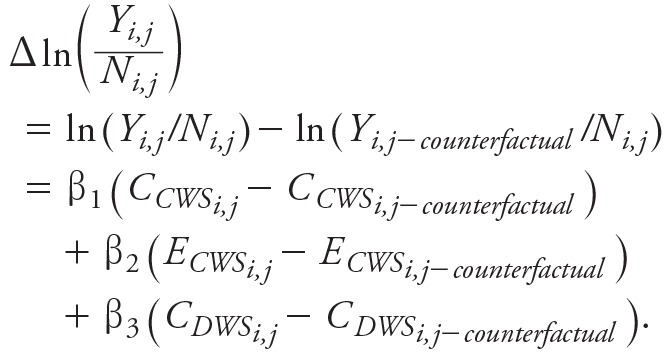
_[3]_

For each county, we summed the estimates of prevented cases across months for each data year to develop annual estimates of avoided cases by county. We then averaged these annual estimates across the 7 years for which ED data were available, correcting for the fact that only 10 months of data were available for 2013.

### ED Visit Costs

To estimate the potential costs associated with ED visits for AGI, we employed cost data from the Medical Expenditure Panel Survey (MEPS), the largest source of healthcare expenditure data available in the United States (Agency for Healthcare Research and Quality 2015). MEPS data are collected annually from a large-scale survey of U.S. households conducted by the Agency for Healthcare Research and Quality of the U.S. Department of Health and Human Services. According to MEPS, the mean and median costs of an ED visit in the southern United States in 2012 (the most recent year for which compiled data are available) were 1,366 USD and 740 USD, respectively (Agency for Healthcare Research and Quality 2012). To represent the potential cost range, we modeled ED visit costs as lognormally distributed with a geometric mean of 740 USD and a geometric standard deviation of 3.03 USD.

## Results

### Microbial Contaminants in North Carolina Drinking Water

Summary statistics for the compiled CWS and private well data show that microbial contaminants are much more common in private wells than in CWSs. Among private wells, 35.7% of the 16,138 samples collected during 2009–2013 tested positive for total coliforms, and 1.37% tested positive for *E. coli*. In comparison, 0.421% of 497,203 CWS samples collected during 2007–2013 tested positive for total coliforms, and 0.0881% of 72,631 samples were positive for *E. coli*. On average, 1.48% of the population in any given county was exposed to total coliform bacteria via a CWS in any given month, whereas 11.7% of the county population was exposed via a private well (Table 1), even though CWS customers outnumber private well users in most counties (see Figure S1). Exposures varied widely across the state (see Figures S2 and S3). Population proportions exposed to contaminants in private wells tended to be higher in the western, Mountain region (see Figure S2) because of the greater reliance on private wells, whereas exposure to CWS contaminants was more common in the Coastal Plain (see Figure S3).

**Table 1:** Summary statistics for the key variables included in the regression model (*n *= 8,200 county-months).

Variable	Mean (SD)	Minimum	First quartile	Median	Third quartile	Maximum
County population (*N*_*i,j*_)	95,355 (141,743)	4,407	24,628	55,622	106,913	919,628
Reported emergency department visits for acute gastrointestinal illness per 1,000 people per month	3.61 (1.84)	0.164	2.25	3.28	4.64	13.5
Percent of county population exposed to total coliform bacteria via private wells (*C*_*DWSi*_)	11.7 (7.78)	0.622	5.02	10.8	16.5	32.1
Percent of county population exposed to a monthly violation of regulations on total coliform bacteria in community water systems (*C*_*CWSi,j*_)	1.48 (8.66)	0.00	0.00	0.00	0.00	95.8
Percent of county population exposed to an acute violation of regulations on *E. coli* bacteria in community water systems (*E*_*CWSi,j*_)	0.0884 (2.23)	0.00	0.00	0.00	0.00	95.5
Percept of population living in poverty (*Pov*_i_)	16.7 (4.52)	8.01	13.5	16.1	19.90	29.0
Has an emergency department (*ED*_*i*_)
Yes	83	NA	NA	NA	NA	NA
No	17	NA	NA	NA	NA	NA
> 16% of residents uninsured (binary) (*I*_*i*_)
Yes	83	NA	NA	NA	NA	NA
No	17	NA	NA	NA	NA	NA
Region
Coastal Plain	41	NA	NA	NA	NA	NA
Piedmont	42	NA	NA	NA	NA	NA
Mountain	17	NA	NA	NA	NA	NA
NA, not applicable.						

### ED Visits for AGI

An average of 405,000 (SD = 38,500) AGI ED visits per year was reported in North Carolina between 2007 and 2013. The overall rate of AGI ED visits from all causes varied substantially across the state and with time (Table 1; see also Figure S4). The average number of monthly visits across all the county-months of available data was 3.61 per 1,000 people (equivalent to 43.3 visits per 1,000 people per year) but ranged from 0.164 to 13.5 per 1,000 people per month (1.96 to 162 visits per 1,000 people per year) (Table 1). Across counties, the number of visits averaged over all months ranged from a low of 1.17 per 1,000 people per month (14 visits per 1,000 people per year) to a high of 8.83 (106 visits per 1,000 people per year) (see Figure S4).

### Associations Between ED Visits for AGI and Regression Model Covariates

**Figure 1:**
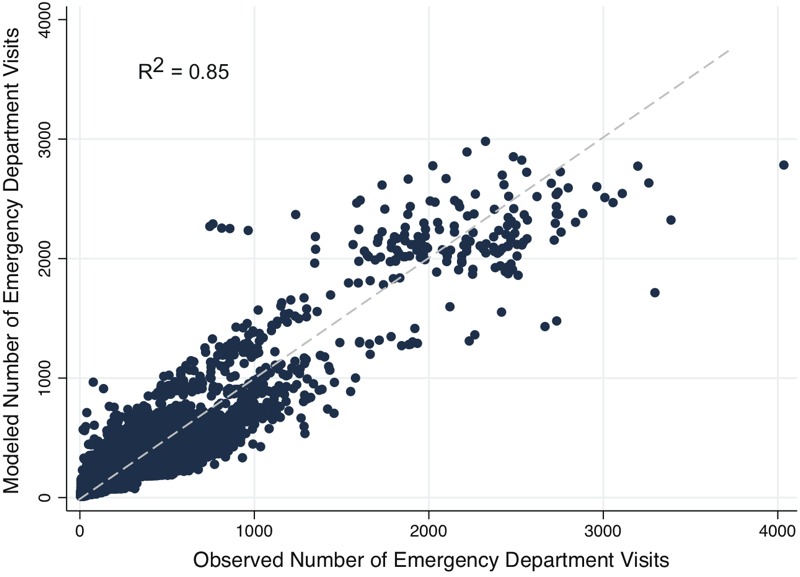
Observed and predicted number of emergency department visits for acute gastrointestinal illness, illustrating the fit of the natural log–Poisson regression model.

The longitudinal multivariate regression model (Equation 1) showed that ED visits for AGI in North Carolina counties were significantly associated with water quality characteristics (Table 2 and Figure 1). ED visits for AGI increased with the prevalence of total coliform bacteria in private wells along with the fraction of the county population exposed to microbial contaminants in community water systems in any given month.

**Table 2:** Beta coefficients from natural log–Poisson regression model fitted to monthly county-level emergency department and water quality data.

Variable	β (95% CI)
Fraction of county population exposed to total coliform bacteria via private wells (*C*_*DWSi*_)	0.844 (0.767, 0.921)
Fraction of county population exposed to a monthly violation of regulations on total coliform bacteria in community water systems (*C*_*CWSi,j*_)	0.00737 (0.00390, 0.0108)
Fraction of county population exposed to an acute violation of regulations on *E. coli* bacteria in community water systems (*E*_*CWSi,j*_)	0.0599 (0.0520, 0.0678)
Fraction of the county population living in poverty (*Pov*_*i*_)	2.57 (2.44, 2.70)
Presence of an emergency department (binary) (*ED*_*i*_)	0.102 (0.0714, 0.132)
Greater than 16% of population uninsured (binary) (*I*_*i*_)	–0.271 (–0.286, –0.255)
Region
Coastal Plain	Referent
Piedmont	–0.111 (–0.124, –0.0990)
Mountain	–0.495 (–0.519, –0.471)
Month
January	Referent
February	0.0285 (0.0267, 0.0303)
March	0.0996 (0.0972, 0.102)
April	–0.0811 (–0.0840, –0.0783)
May	–0.131 (–0.134, –0.127)
June	–0.188 (–0.192, –0.185)
July	–0.181 (–0.185, –0.178)
August	–0.173 (–0.177, –0.170)
September	–0.180 (–0.183, –0.176)
October	–0.164 (–0.167, –0.161)
November	–0.158 (–0.161, –0.155)
December	–0.0377 (–0.0397, –0.0357)
Constant (α)	–5.94 (–5.98, –5.90)
CI, confidence interval.	

The regression model results also highlight other important influences on rates of ED visits for AGI. Poverty has an important influence. The β coefficient on poverty (2.57) implies that an average of 0.25 additional ED visits for AGI per 1,000 people per month occurred in counties in the highest quartile of statewide poverty (19.9% living in poverty) than in counties in the lowest quartile (13.5%) after adjusting for measures of drinking water quality. In addition, as suggested in Figure S4 and as demonstrated by the regression coefficients in Table 2, AGI ED visit rates were significantly higher in the Coastal Plain region than in the other two regions and were lowest in the Mountain Region. ED visit rates were lower in counties where more people were uninsured and higher in counties with EDs, as indicated by the negative and positive signs on the regression coefficients for these variables. Seasonally, AGI visit rates were highest in winter (December through February), as indicated by the negative coefficients on nonwinter months.

### ED Visits Attributable to Domestic Well Contamination

Employing this regression model in the PIM analysis suggests that an estimated 29,400 (95% CI: 26,600, 32,200) ED visits for AGI were attributable to microbial contamination in drinking water each year, constituting approximately 7.3% (95% CI: 6.6, 7.9%) of all ED visits for AGI (Table 3, top row). Approximately 99% of the attributable visits (29,200; 95% CI: 26,500, 31,900) were associated with private well contamination, and the remaining 1% were associated with CWS contamination (Table 3, top row). The PIM approach estimates that if 10% of the population relying on private wells in each county were connected to a local CWS, then 2,920 (95% CI: 2,650, 3,190) ED visits for AGI could be prevented across North Carolina each year (Table 3, top row).

**Table 3:** Emergency department visits for acute gastrointestinal illness attributable to microbial contamination of drinking water in North Carolina and associated costs under alternative scenarios.

Scenario	ED visits attributable to drinking water contamination (number/year)	ED visits attributable to private well contamination (number/year)	Cost of ED visits attributable to private well contamination (millions USD/year)	ED visits preventable by extending water service to 10% of private well population (number/year)	Value of ED visits preventable by extending water service to 10% of private well population (millions USD/year)
Best estimate	29,400 (26,600–32,200)	29,200 (26,500–31,900)	39.9 (2.56–192)	2,920 (2,650–3,190)	3.99 (0.256–19.2)
Alternative estimate 1	27,600 (25,000–30,200)	27,600 (25,000–30,200)	37.7 (2.21–180)	2,740 (2,480–2,990)	3.77 (0.221–18.0)
Alternative estimate 2	31,300 (28,400–34,200)	31,300 (28,400–34,200)	42.4 (2.52–198)	3,110 (2,820–3,390)	4.23 (0.251–19.8)
ED, Emergency department. Alternative estimates 1 and 2 were derived by assuming that the prevalences of total coliform bacteria in private wells in each of the nine counties that did not provide private well data were equal to the 15th and 85th percentile values of the statewide prevalence, respectively. The nine counties for which data were missing were Buncombe, Caldwell, Catawba, Cherokee, Cleveland, Gaston, Haywood, New Hanover, and Wake.					

**Figure 2:**
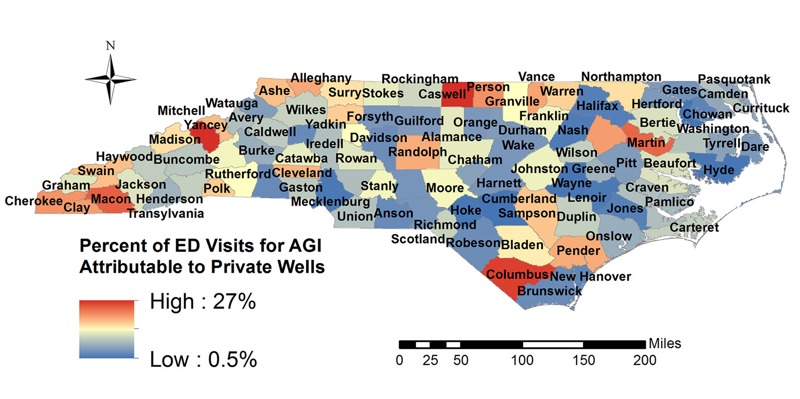
Estimated percentage of emergency department (ED) visits for acute gastrointestinal illness (AGI) attributable to private wells [Map data, Minnesota Population Center (2011)].

**Figure 3:**
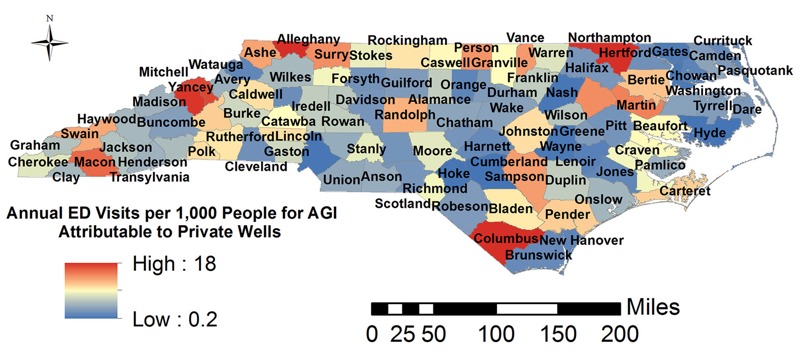
Estimated annual rate of emergency department (ED) visits per 1,000 people for acute gastrointestinal illness (AGI) attributable to private wells [Map data, Minnesota Population Center (2011)].

The health burden associated with microbial contamination of domestic wells varies substantially by county. The proportion of AGI ED visits potentially attributable to DWSs ranges by county from 0.525% to 27.1% (Figure 2). County-level rates of attributable AGI visits per 1,000 people per year range from 0.179 to 17.7 (Figure 3).

### Costs of ED Visits Attributable to Domestic Well Contamination

The estimated state-wide cost of ED visits for AGI attributable to microbial contamination in drinking water was 40.2 million USD (95% CI: 2.58 million USD, 193 million USD). Of this total, 39.9 million USD (95% CI: 2.56 million USD, 192 million USD) was estimated to arise from private well contamination (Table 3). Extending community water service to 10% of the population in each county currently relying on private wells would decrease annual AGI ED visit costs by 3.99 million USD (95% CI: 256,000 USD, 19.2 million USD). The total net present value of this potential benefit over 30 years, assuming a 3% discount rate (the approximate current interest rate for municipal bonds), is 78.1 million USD (95% CI: 5.01 million USD, 376 million USD).

### Sensitivity Analysis

Because data on the prevalence of microbial contaminants in private wells were unavailable for 9 of North Carolina’s 100 counties, we analyzed the sensitivity of the estimated disease burden associated with private well contamination to alternative assumptions about well water quality in these nine counties. Our best estimate (Table 3, top row) assumed that the prevalence of private well contamination in each of these counties was equal to the mean of the prevalences in the surrounding counties. Alternative estimates 1 and 2 (Table 3, bottom rows) assumed that the prevalences of private well contamination in each of these nine counties were equal to the 15th and 85th percentile values, respectively, of prevalences in the state as a whole. Overall, these changes had a small effect on our results, changing the baseline estimates by approximately ± 6% (Table 3).

## Discussion

We estimated that approximately 7.3% (95% CI: 6.6, 7.9%) of all ED visits for AGI from 2007 to 2013 were potentially attributable to microbial contamination of North Carolina drinking water. Approximately 99% of the attributable cases were associated with contamination in private wells, according to our estimates. On average, ED visits potentially attributable to private well contamination are estimated to cost 39.9 million USD per year.

### Comparison with Prior Research

To our knowledge, there have not been any previous assessments of AGI risk due to private wells in the United States; the closest equivalent studies on drinking water quality we could find in the literature were two studies of nondisinfected groundwater. Using a quantitative microbial risk assessment approach, Macler and Merkle (2000) estimated that microbial contamination of nondisinfected community groundwater systems contributed to 0.75–5.9 million AGI cases annually in the United States (5–39% of all cases among the population using nondisinfected community groundwater systems). Borchardt et al. (2012) found that 6–22% of self-reported AGI cases were attributable to viruses in tap water in 13 Wisconsin communities that did not disinfect their community groundwater supplies. Our estimate that 7.3% of AGI cases seen in North Carolina EDs were attributable to contaminated private wells is on the low end of estimates reported in studies of nondisinfected groundwater CWS studies.

These results lend support to the value of total coliform bacteria as indicators of public health risk for private wells. Our results show that for a county in which 35% of the population relied on private wells (the statewide average), every 10% increase in the prevalence of total coliform bacteria in private wells increased the countywide number of ED visits for AGI by 3.0%, controlling for demographic factors. Although researchers have long sought improved indicators of pathogens in drinking water (e.g., Savichtcheva and Okabe 2006), these results suggest that continued monitoring of private wells for total coliform bacteria can provide valuable information on the public health risks of private well contamination. This finding supports results from a recent review of 20 years of research on pathogens in water by Payment and Locas (2011). Specifically, Payment and Locas noted, “Quite interestingly, in our studies of groundwater…, it was the nonfecal indicators, total coliforms, and aerobic endospores that were found most frequently in virus-positive samples.” Payment and Locas reported that *E. coli* and *Enterococci*, which are more-specific indicators of fecal contamination than total coliforms, were absent in 20% and 30% of groundwater samples testing positive for culturable human enteric viruses, respectively, whereas total coliform bacteria were positive in all virus-positive samples. Payment and Locas concluded,

The presence of total coliforms in groundwater indicates that microorganisms from surface water have been able to reach the aquifer and a more rigorous monitoring should begin for other microorganisms (pathogenic) which might also reach the aquifer. When fecal indicators are detected, anything can happen, and will happen, with potential serious public health implications.

### Domestic Wells in Peri-Urban Areas

Although most North Carolina communities lacking regulated water service are located in rural areas, particularly in the mountainous western part of the state (see Figure S1), some are located in relatively population-dense neighborhoods on the fringes of, or entirely surrounded by, cities and towns served by CWSs (Naman and MacDonald Gibson 2015; MacDonald Gibson et al. 2014). In some cases, these communities were historically denied access to municipal services during the era of legally sanctioned racial segregation and still have not received access to services (Dewan 2005; Gilbert 2013; Johnson et al. 2004; Marsh et al. 2013). A handful of community-level case studies documenting such disparities exist (Johnson et al. 2004; MacDonald Gibson et al. 2014; UNC Center for Civil Rights 2006). One example is a neighborhood adjacent to Mebane, a town with a population of ~8,000 located 50 mi (80 km) northwest of Raleigh. Recently, as a result of more than a decade of action by a local community organization, Mebane extended CWS services to 90 homes, but > 400 homes remain without service (Heaney et al. 2011; Wilson 2011). Such population-dense areas near existing infrastructure may be the most appropriate targets for future CWS expansion because of the likely relatively lower cost (compared with rural areas) of extending existing water distribution networks.

Local governments and utility providers traditionally make decisions pertaining to water service, and a large portion of these decisions are made on a cost–benefit basis. Constructing water mains is expensive, and it is not feasible to provide regulated water statewide. However, identifying areas of greater population density that may be in close proximity to existing infrastructure and factoring in the potential health benefits may make expansion economically feasible. Future research should identify such communities.

### Limitations

A number of limitations are inherent in the data analyzed in this study. First, owing to a lack of pathogen monitoring, we relied on the presence of total coliform bacteria as the indicator of potential exposure to microbial pathogens because these data are routinely collected by CWSs and are also collected when new private wells are constructed. Such microbial indicators are used for reasons of practicality and cost because large water samples are required to detect pathogens, and sampling techniques are costly (e.g., for *Giardia* and viruses) (Hancock et al. 1998; Macler and Regli 1993; Regli et al. 1991). The presence of a microbial indicator does not confirm but rather increases the probability of pathogen presence; likewise, the absence of indicator organisms does not guarantee that the water is pathogen-free (Payment and Locas 2011). Therefore, our understanding of the presence of pathogens is conditional on the indicator organism, so we may have over- or underestimated exposure (Borchardt et al. 2012; Johnson et al. 2011; Kay et al. 2007; Payment and Locas 2011). Potentially amplifying this effect is that the private well water quality samples were collected upstream of in-home treatment systems, where such systems were in use. In contrast, community water systems collect water samples after treatment. In-home devices can increase, decrease, or have no effect on levels of microbial contamination. For example, Chaidez and Gerba found that in-home activated carbon filters “may amplify the numbers of bacteria present in the tapwater by promoting biofilm formation” (Chaidez and Gerba 2004). Although reverse-osmosis, distillation, and disinfection systems can remove microbial contaminants, previous studies suggest that the prevalence of use of such devices is relatively low among private well owners. For example, a survey of 221 private well owners in Michigan found that 8.6% used a home treatment device capable of removing microbial contaminants (Slotnick et al. 2006).

A second limitation is the assumed uniform exposure across the population served by each CWS for a given month with a violation and the similar uniform exposure assumed for private wells within a county within the time period analyzed. These assumptions could result in under- or overestimates of the number of people exposed if the proportion of private well users exposed to microbial indicator organisms in a given county was not constant over the course of the analysis time period, or if the CWS population was not uniformly exposed during a given month. Exposure may be overestimated if some residents exclusively drink bottled water. Additionally, all new well owners receive public health recommendations to disinfect their wells and/or install treatment systems if the well tests positive for contamination and as a result may take corrective action, which would reduce exposure levels; such corrective actions are not reflected in this analysis. By contrast, underestimates of exposure could have occurred if private well water quality deteriorated after construction. Our private well data set included only newly constructed wells, which may not be representative of older private wells with aging components. Similarly, we could have underestimated exposure to CWS contamination because exposure for CWSs was defined as an MCL violation (> 5% of samples testing positive in a given month), whereas in fact, exposure may still occur when < 5% of samples test positive.

A third limitation arises from the geopolitical level of the analysis. The finest resolution of NC DETECT’s data on AGI ED visits made available for this research was at the county level. Therefore, we assumed a homogeneous distribution of AGI across each county and, as a result, may have introduced bias in our estimates. In addition, as a result of the county-level aggregation of the health outcome data, exposure estimates also needed to be expressed at the county level. Exposure due to a given water system type (private well or CWS) at the county level was estimated using a population weighting approach. Thus, the contribution of CWSs to the risk estimates was in proportion to their population size, whereas private wells were assumed to have a uniform size across all systems in the county. Further, the aggregation of CWS exposure at the county level has the potential to be biased owing to the influence of larger systems. These assumptions were unavoidable given the nature of reported data on microbial indicator organisms.

A fourth limitation arises from the way in which ED visit data are coded. Patient data are classified based on ICD-9 codes, which are used for billing rather than for diagnosis; thus, they may contribute to under- or overestimation of the true health risk. Underestimation may occur when two or more conditions are present during a visit and medical personnel elect to report the more severe or more important billing code, neglecting to mention the AGI that was in fact present. Overestimation may occur as a result of the general coding protocols of an ED and the assumption of which comorbidities are present for a given condition.

A final caveat is that because our study involved neither random sampling nor random allocation, results may be due to the factors under investigation, unmeasured factors, or measurement error, but not chance (Greenland 1990). Because of these possibilities, caution should be taken in interpreting the statistical significance of the PIM.

Overall, the estimates presented herein likely underestimate the total health burden resulting from microbial contamination of domestic wells. The health outcome data set captures only a fraction of all AGI cases. A previous study based on phone surveys of 52,840 people across the United States estimated that 6.4% (95% CI: 5.0, 7.8%) of persons with AGI visit an ED (Jones et al. 2007). Thus, every ED visit potentially represents ~16 (= 1/0.064) AGI cases.

## Conclusions

Despite its limitations, this analysis demonstrates a new method for estimating waterborne disease risks associated with lack of community water service that could be applied not only in the United States and other developed nations but also in developing countries, as was recently recommended by Clasen et al. (2014). In the United States, concerns about disparities in water service levels have been reported recently in communities ranging from Alaska Native villages to agricultural areas in central California to the Southeast (Balazs and Ray 2014). The method demonstrated in this paper could be used to quantify the public health implications of these disparities.

Historically, public health practitioners have played a critical role in persuading municipalities to adopt water treatment systems. Our finding that some 29,200 annual ED visits for AGI costing ~39.9 million USD are potentially attributable to contamination of private wells indicates that expanding regulated water services has the potential to provide substantial health benefits. Where service extensions are not technically or economically feasible, county or state governments could expand services to support private well owners in maintaining the integrity of their wells, routinely testing their water quality, and, where necessary, installing and maintaining in-home treatment. Public health practitioners could use the information in this analysis to encourage a new dialogue with local water utilities and governments about options for extending municipal water service into unserved areas and for providing other support measures where such extensions are not feasible.

## Supplemental Material

(2.2 MB) PDF
