# Supplementary material for: Reducing Emergency Department Visits for Acute Gastrointestinal Illnesses in North Carolina (USA) by Extending Community Water Service
Source: Environ Health Perspect. 2016 May 20;124(10):1583–91. doi: 10.1289/EHP160 (PMC5047767; doi:10.1289/EHP160)
Supplement: (2.2 MB) PDF [file EHP160.s001.acco.pdf]

Environ Health Perspect
DOI: 10.1289/EHP160

Note to readers with disabilities: *EHP* strives to ensure that all journal content is accessible to all readers. However, some figures and Supplemental Material published in *EHP* articles may not conform to 508 standards due to the complexity of the information being presented. If you need assistance accessing journal content, please contact ehp508@niehs.nih.gov. Our staff will work with you to assess and meet your accessibility needs within 3 working days.

Supplemental Material

Reducing Emergency Department Visits for Acute Gastrointestinal Illnesses in North Carolina (USA) by Extending Community Water Service

Nicholas B. DeFelice, Jill E. Johnston, and Jacqueline MacDonald Gibson

Table of Contents

Figure S1. Percent of county population relying on private wells for drinking water.
Figure S2 Percent of county population exposed to microbiological drinking water contaminants via private wells (average for the time period January 1, 2009–December 31, 2013).
Figure S3. Percent of county population exposed to a violation of the maximum contaminant level (MCL) for microbiological contaminants in community water systems (monthly average for the time period January 1, 2007–December 31, 2013).
Figure S4. Annual county rate of emergency department (ED) visits for acute gastrointestinal illness (AGI)**.**

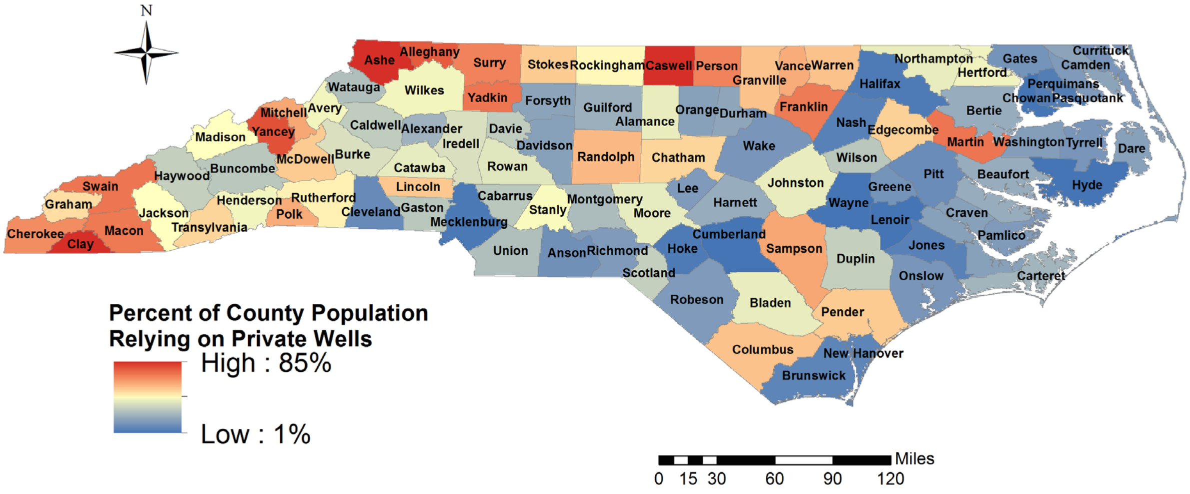

Figure S1. Percent of county population relying on private wells for drinking water.

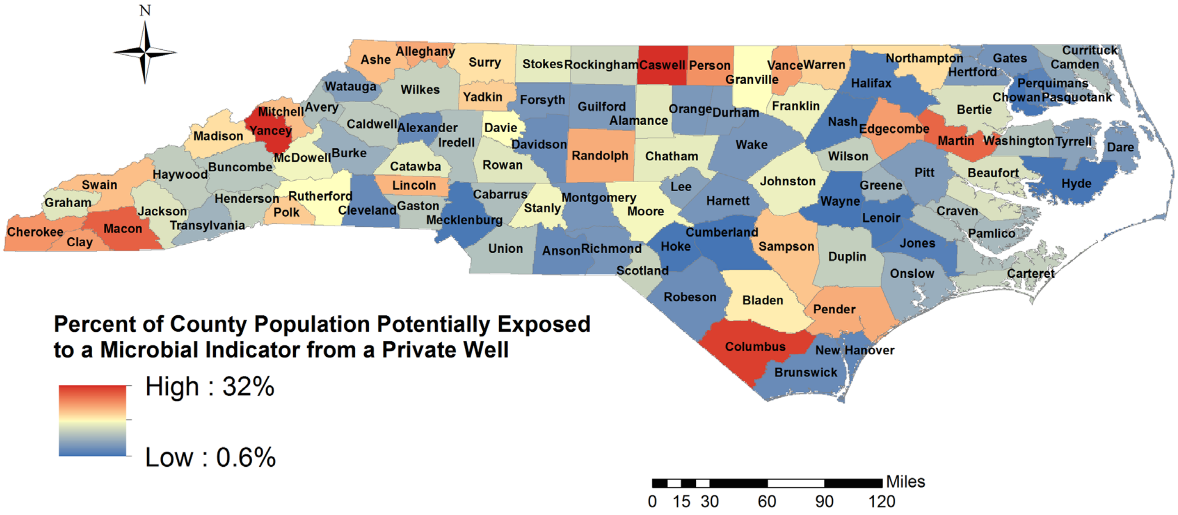

Figure S2. Percent of county population exposed to microbiological drinking water contaminants via private wells (average for the time period January 1, 2009–December 31, 2013).

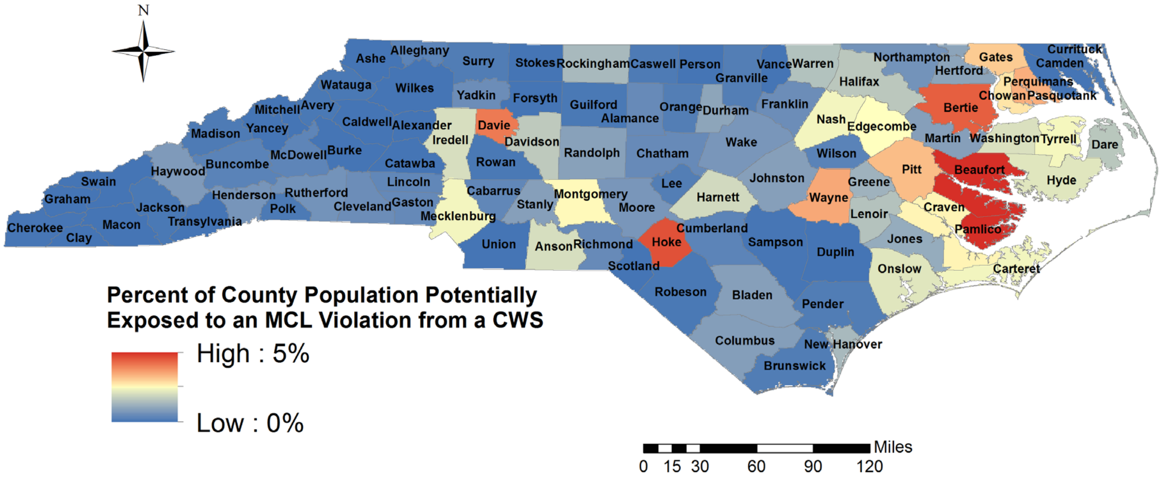

Figure S3. Percent of county population exposed to a violation of the maximum contaminant level (MCL) for microbiological contaminants in community water systems (monthly average for the time period January 1, 2007–December 31, 2013).

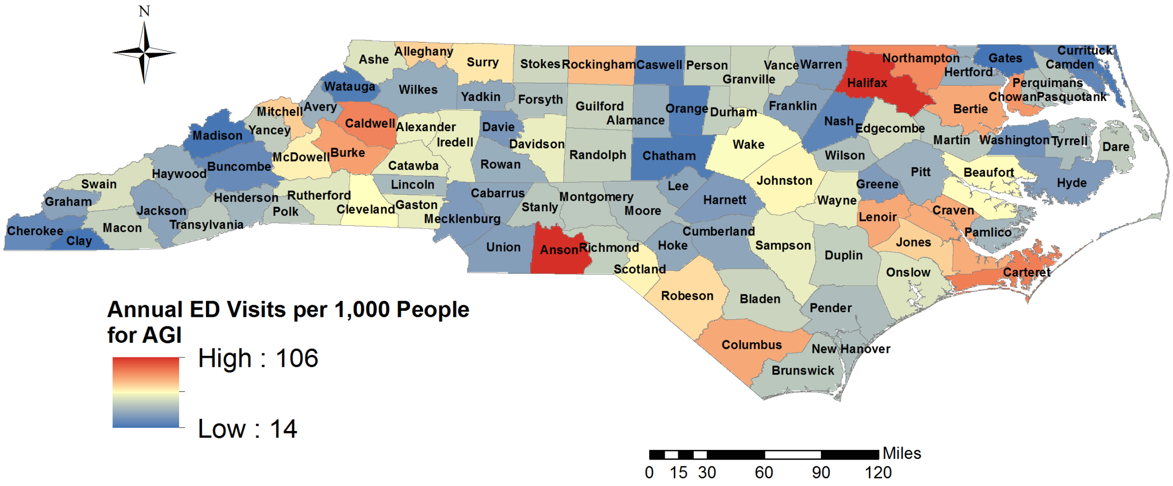

Figure S4. Annual county rate of emergency department (ED) visits for acute gastrointestinal illness (AGI).
